# Tubuloside A, a phenylethanoid glycoside, alleviates diclofenac induced hepato‐nephro oxidative injury via *Nrf2/HO‐1*

**DOI:** 10.1111/jcmm.17968

**Published:** 2023-09-29

**Authors:** Ali Tureyen, Hasan Huseyin Demirel, Ezgi Nur Demirkapi, Azra Mila Eryavuz, Sinan Ince

**Affiliations:** ^1^ Department of Gastroenterology Ministry of Health Eskisehir City Hospital Eskisehir Turkey; ^2^ Bayat Vocational School Afyon Kocatepe University Afyonkarahisar Turkey; ^3^ Faculty of Veterinary Medicine, Department of Physiology Afyon Kocatepe University Afyonkarahisar Turkey; ^4^ Department of Biochemistry, Faculty of Veterinary Medicine Afyon Kocatepe University Afyonkarahisar Turkey; ^5^ Department of Pharmacology and Toxicology, Faculty of Veterinary Medicine Afyon Kocatepe University Afyonkarahisar Turkey

**Keywords:** apoptosis, diclofenac, inflammation, oxidative stress, Tubuloside A

## Abstract

The most prominent adverse effects of nonsteroidal anti‐inflammatory drugs (NSAIDs) such as diclofenac (DF) are hepato‐renal damage. Natural antioxidants can be preferred as an alternative and/or combination to improve this damage. This present study was conducted to evaluate the protective effect of Tubuloside A (TA) against diclofenac (DF)‐induced hepato‐renal damage. TA (1 mg/kg, ip) was administered to male Sprague–Dawley rats for 5 days, and DF (50 mg/kg, ip) was administered on Days 4 and 5. Plasma aspartate amino transferase, alanine amino transferase, alkaline phosphatase, blood urea nitrogen and creatinine were measured to evaluate liver and kidney functions. Additionally, oxidative stress parameters (malondialdehyde, glutathione, superoxide dismutase, catalase, and 8‐oxo‐7,8‐dihydro‐2′‐deoxyguanosine) in blood, liver, and kidney tissues, changes in mRNA expression of genes involved in the *Nrf2*/*HO*‐*1* signalling pathway (*Nrf2*, *HO*‐*1*, *NQO*‐*1*, *IL*‐*6*, *iNOS*, *Cox*‐*2*, *TNF*‐*α*, *IL1*‐*β* and *NFκB*) and apoptotic process (*Bcl*‐*2*, *Cas*‐*3* and *Bax*) in liver and kidney tissues were determined. Additionally, tissue sections were evaluated histopathologically. Biochemical, histopathological, and molecular results demonstrated the hepato‐renal toxic effects of DF, and TA treatment protected the liver and kidney from DF‐induced damage. This provides an explanation for the hepato‐nephro damage caused by DF and offers new ideas and drug targets together with TA for the prevention and treatment of DF injury.

## 1 INTRODUCTION

Diclofenac (DF) is a nonsteroidal anti‐inflammatory drug (NSAID) with widespread use in the treatment of osteoarthritis and rheumatoid pain, in addition to skeletal system injuries characterized by lipophilic.^1^ As is known, NSAID drugs inhibit cyclooxygenase (*Cox*), leading to the suppression of thromboxane and prostaglandin synthesis.^2^ While the ordinary therapeutic uses of DF are considered safe, their use at long intervals or at high doses causes bleeding and ulcerations in the digestive system, along with liver and kidney damage.^3^, ^4^ DF is metabolized by multiple cytochrome P‐450 enzymes in hepatocytes, leading to drug‐protein binding, glutathione (GSH) conjugation and mitochondrial dysfunction, which cause organ damage.^5^

Co‐treatment of a proton pump inhibitor is clinically preferred to minimize NSAID‐induced gastrointestinal damage.^6^ However, some studies have shown that these gastroprotective drugs synergistically exacerbate NSAID‐induced intestinal damage and bleeding.^7^ In addition, glucocorticoids are preferred for the treatment of NSAID‐induced acute renal failure.^8^ Therefore, new therapeutic agents are needed to mitigate both NSAID‐induced gastrointestinal damage and renal damage caused by glucocorticoid therapy.

The *Cistanche tubulosa* plant, which contains many phenylethanoid glycosides, including Tubuloside A (TA), is widely used by the public for the treatment of forgetfulness, impotence and constipation.^9^ Particularly in Southeast Asian countries, *C*. *tubulosa* extract is used as a dietary supplement for anti‐aging, neuroprotective, antidepressant, or antiarrhythmic purposes and metabolic diseases.^10^, ^11^, ^12^, ^13^ In experimental studies, it has been reported that *C*. *tubulosa* extract inhibits noradrenaline‐induced contractions in isolated rat aortic strips,^14^ exhibits hepatoprotective effects against d‐galactosamine (d‐GalN)/lipopolysaccharide‐induced liver damage in mice,^15^ and shows hypoglycemic and hypolipidemic effects in rodents with induced diabetes.^16^, ^17^ Also, some research suggested that silymarin (SL) exhibits a hepato‐nephro protective effect because of its radical scavenger effects.^18^, ^19^

As far as we know, there is no literature on the protective role of TA on hepato‐renal toxicity caused by DF. Therefore, in this study, the clinical potential of TA to counteract hepato‐renal toxicity accompanying DF chemotherapy was investigated. In this regard, the effect of administering DF and TA to rats was determined by changes in biochemical and oxidative stress parameters, histopathological examinations of liver and kidney tissues, as well as alterations in mRNA expressions of apoptotic and inflammatory cytokines in these tissues.

## 2 MATERIALS AND METHODS

### 2.1 Chemicals

The DF used in the study was obtained from DEVA Pharmaceuticals Inc., TA was obtained from ChemFaces (CAS number: 112516‐05‐9), and SL was obtained from Sigma‐Aldrich (CAS number: 65666‐07‐1). Other chemicals used were purchased from commercial suppliers.

### 2.2 Experimental design

Thirty 4–6‐week‐old male Sprague–Dawley rats (200 ± 25 g) were obtained from the Animal Research and Application Unit of Afyon Kocatepe University. The study was conducted in accordance with universal ethical principles and with the approval of the local ethics committee (49533702/160). Rats were kept in a standard room with a 12‐h light/dark cycle at a temperature of 21 ± 2°C and a humidity of 50%–60%. The rats were randomly divided into five groups, each consisting of 6 animals, and prior to the experiment, all rats were acclimatized for 7 days. The groups were negative control (no treatment), intraperitoneal injection of 1 mg/kg TA,^12^ oral treatment of 50 mg/kg DF sodium,^20^ DF sodium plus TA, and DF sodium plus oral treatment of 25 mg/kg SL. SL was used as a positive control to compare the efficacy of TA.^19^ TA and SL were administered for 5 days, while DF was given once on the fourth and fifth days, 1 h after the treatment of TA and SL. Twenty‐four hours after the last treatment, the rats were anaesthetised with isoflurane, and their liver and kidney tissue samples were collected by the intracardiac puncture. A portion of the liver and kidney tissues was homogenized with 0.15 M Tris‐HCl buffer (pH 7.4) and used for biochemical analyses. Tissue samples for molecular analyses were frozen in liquid nitrogen and stored at −80°C until analysis. Histopathological tissue samples were placed in 10% formaldehyde solution and processed in the laboratory.

### 2.3 Determination of biochemical parameters

Measurement of aspartate aminotransferase (AST), alanine aminotransferase (ALT), alkaline phosphatase (ALP), blood urea nitrogen (BUN) and creatinine in rat plasma was performed using commercial kits from BIOLABO (Medica) obtained by spectrophotometry. In addition, measurement of 8‐oxo‐7,8‐dihydro‐2′‐deoxyguanosine (8‐OHdG) in plasma, liver and kidney tissues was performed using ELISA kits (Elabscience). Spectrophotometric measurements were conducted using a Shimadzu 1601 UV–VIS spectrophotometer.

### 2.4 Determination of lipid peroxidation and antioxidant enzyme activities

The level of malondialdehyde (MDA), a marker of lipid peroxidation, was measured in whole blood and tissues according to the methods proposed by Draper and Hardley^21^ and Ohkawa et al.,^22^ respectively. Glutathione (GSH) levels in whole blood and tissues were determined using the method of Beutler et al.,^23^ while superoxide dismutase (SOD) and catalase (CAT) activities in erythrocyte lysates and tissues were determined using the methods of Sun et al.^24^ and Sinha,^25^ respectively. Haemoglobin levels in erythrocyte lysates and protein amounts in tissues were determined using the methods revealed by Drabkin and Austin^26^ and Lowry et al.,^27^ respectively. The measurements of these analyses were performed spectrophotometrically using Shimadzu 1601 UV–VIS.

### 2.5 Determination of mRNA expression levels

Total RNA was isolated from liver and kidney tissues using the A.B.T.™ Blood/Tissue RNA Purification Kit (Atlas Biotechnology). The quality of RNA was determined using the Multiskan™ FC Microplate Photometer (Thermo Scientific). RevertAid H Minus Single‐Strand cDNA Synthesis Kit (Thermo Scientific) was used for cDNA synthesis from RNA. MRNA sequences of genes specific to *Rattus norvegicus* were obtained from the NCBI website and were named using the computer software package Fast PCR 6.0. Primers were obtained from Sentegen Biotechnology and are shown in Table 1. *Nrf2*, *HO*‐*1*, *NQO*‐*1*, *Bcl*‐*2*, *Cas*‐*3*, *IL*‐*6*, *iNOS*, *Cox*‐*2*, *Bax*, *TNF*‐*α*, *IL1*‐*β* and *NFκB* mRNA levels were determined using RT‐PCR (StepOnePlus, Applied Biosystems). Each sample was analysed in triplicate and normalized to the expression level of the housekeeping gene *β*‐*actin*. The results were expressed as relative gene expression using the delta–delta CT method (^28^).

**TABLE 1:** Primers, oligonucleotide sequences and product size of genes.

Primer	Sequence	Product size (bp)
β‐Actin	F: AGCAAGAGAGGCATCCTCACC	21
	R: ACAGGGATAGCACAGCCTGGA	21
Bcl‐2	F: GGTGAACTGGGGGAGGATTG	20
	R: AGAGCGATGTTGTCCACCAG	20
NQO‐1	F: AGGAAACAGCAGCCAAGGTA	20
	R: AGGAAACAGCAGCCAAGGTA	20
Nrf2	F: TTTGTAGATGACCATGAGTCGC	22
	R: TGTCCTGCTGTATGCTGCTT	20
HO‐1	F: GCCTGGTTCAAGATACTACCTCT	23
	R: CTGAGTGTGAGGACCCATCG	20
Cas‐3	F: GGACAGCAGTTACAAAATGGATTA	24
	R:CGGCAGGCCTGAATGATGAAG	21
IL‐6	F: TCTGGTCTTCTGGAGTTCCGT	21
	R: GGAAGTTGGGGTAGGAAGGAC	21
iNOS	F: ATTGGCACCATCTAACGCACT	21
	R: TGGGGATTTTGTTCTGGGCAT	21
Cox‐2	F: GAGTACCGCAAACGCTTCTCC	21
	R: CTTGCGTTGATGGTGGCTGTC	21
Bax	F: AGGACGCATCCACCAAGAAGC	21
	R: CAGTGAGGACTCCAGCCACAA	21
TNF‐α	F: CATCCGTTCTCTACCCAGCC	20
	R: AATTCTGAGCCCGGAGTTGG	20
IL1‐β	F: AGGCTGACAGACCCCAAAAG	20
	R: CTCCACGGGCAAGACATAGG	20
NFκB	F: TGGACGATCTGTTTCCCCTC	20
	R: CCCTCGCACTTGTAACGGAA	20

### 2.6 Determination of histopathological changes

Liver and kidney samples placed in a formaldehyde solution were cut into 2–3 mm thickness and embedded in tissue tracking cassettes. After washing with running tap water overnight, they were blocked in paraffin by being held for 2 h each in alcohol series (50%, 70%, 80%, 96% and absolute alcohol), xylene, xylene‐paraffin and melted paraffin at 56–58°C. Samples with a thickness of 5 μ were taken from the paraffin blocks using a microtome (Leica, RM 2245), and then they were dried for 10 min in an oven (Thermo, Heraterm). All sections were passed through alcohol series of absolute, 96%, 80%, 70% and 50% and stained with haematoxylin–eosin (HE). The stained preparations were examined under a binocular‐headed light microscope (Nikon, Eclipse Ci), and the microscopic images of the preparations were taken with the microscopic digital camera system (Nikon DS Fİ3). The differences among the groups were statistically evaluated according to damage scores as 0: none, 1–2: mild, 3–4: moderate and >4: severe.

### 2.7 Statistical analyses

At the end of the study, the data was analysed using statistical software (SPSS 22 software package). First, it was determined whether the data followed a normal distribution pattern. The data that followed a normal distribution was evaluated using the one‐way anova test, and differences between groups were determined using Duncan's post hoc test. The results were expressed as “mean ± standard deviation” and considered statistically significant if <0.05.

## 3 RESULTS

### 3.1 Effect on biochemical parameters

**FIGURE 1:**
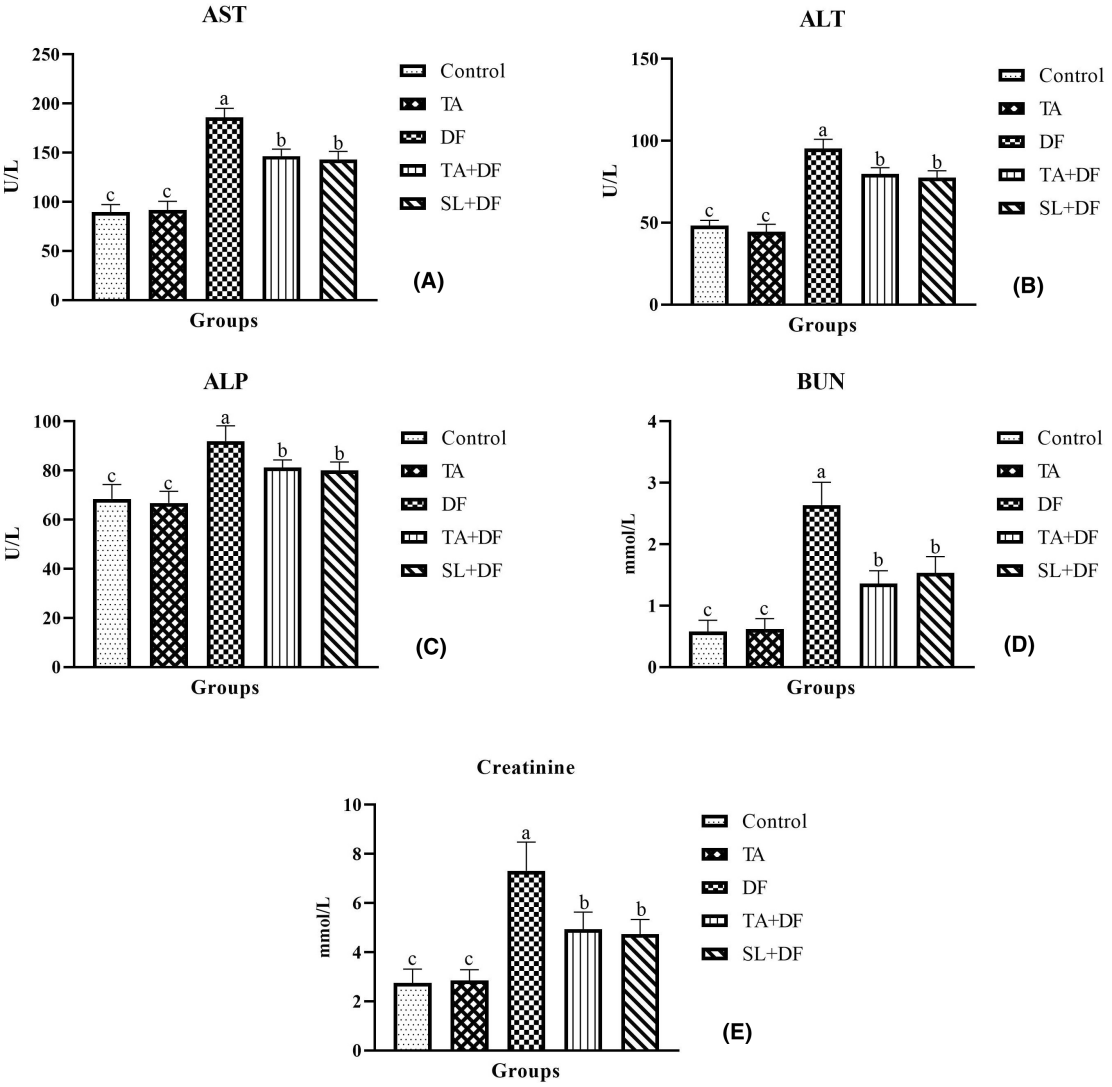
The effect of Diclofenac (DF) and Tubuloside A (TA) on plasma aspartate aminotransferase (AST‐A), alanine aminotransferase (ALT‐B), alkaline phosphatase (ALP‐C), blood urea nitrogen (BUN‐D), and creatinine (E) levels. Mean ± standard deviations; *n*: 6; ^a, b, c^ values with different letters in the figure are statistically significant (*p* < 0.001). DF, diclofenac; TA, tubuloside A; SL, silymarin.

The liver function parameters, AST (Figure 1A), ALT (Figure 1B), and ALP (Figure 1C) activities, and kidney function parameters, BUN (Figure 1D) and creatinine (Figure 1E) values were found high levels compared with the control (*p* < 0.001). However, the TA and SL treatments reduced the increasing values of DF (*p* < 0.001). In addition, the TA treatment alone did not cause any change in these values compared to the control (*p* > 0.05).

### 3.2 Effect on oxidative stress

**FIGURE 2:**
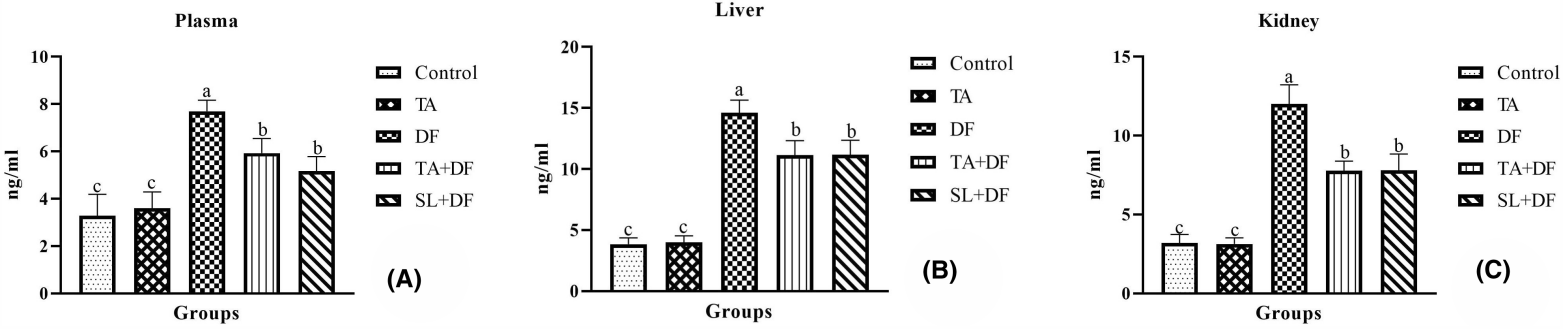
The effect of Diclofenac (DF) and Tubuloside A (TA) on plasma (A), liver (B) and kidney (C) 8‐oxo‐7,8‐dihydro‐2′‐deoxyguanosine (8‐OHdG) levels. Mean ± standard deviations; *n*: 6; ^a, b, c:^ values with different letters in the figure are statistically significant (*p* < 0.001). DF, diclofenac; TA, tubuloside A; SL, silymarin.

8‐OHdG levels in plasma (Figure 2A), liver (Figure 2B) and kidney (Figure 2C) tissues were found high level compared with the control group (*p* < 0.001). However, the TA and SL treatments were found to reduce the increasing 8‐OHdG values with the DF treatment (*p* < 0.001). In addition, the TA treatment alone did not cause any change in 8‐OHdG values compared to the control (*p* > 0.05).

### 3.3 Effect on lipid peroxidation and antioxidant status

DF treatment increased the MDA levels in the rat's whole blood, liver, and kidney tissues compared to the control group, while decreasing the GSH levels in whole blood, liver, and kidney tissues, and SOD and CAT activity levels in erythrocytes, liver, and kidney tissues (Table 2) (*p* < 0.001). However, TA and SL treatments reversed these changes induced by DF and approached the control (*p* < 0.001). Additionally, TA treatment alone did not cause a significant change in these values compared to the control (*p* > 0.05).

**TABLE 2:** The effects of TA and DF administration on malondialdehyde (MDA) and glutathione (GSH) levels or superoxide dismutase (SOD) and catalase (CAT) activities in tissue homogenates.

Parameters	Tissue Homogenates	Groups				
		Control	TA	DF	TA + DF	SL + DF
**MDA**	Blood (nmoL/mL)	1.60 ± 0.08^c^	1.38 ± 0.20^c^	8.28 ± 1.03^a^	5.61 ± 0.86^b^	5.71 ± 1.03 ^b^
	Liver (nmoL/g tissue)	4.00 ± 1.17^c^	3.99 ± 0.93^c^	14.37 ± 1.79 ^a^	9.63 ± 1.25^b^	9.66 ± 1.06^b^
	Kidney (nmoL/g tissue)	3.34 ± 0.80^c^	3.42 ± 0.46^c^	10.26 ± 1.12 ^a^	7.39 ± 0.79^b^	7.46 ± 0.83^b^
**GSH**	Blood (nmoL/mL)	29.82 ± 3.03^a^	29.20 ± 0.91^a^	10.62 ± 1.17^c^	21.41 ± 1.27^b^	23.19 ± 2.75^b^
	Liver (nmoL/g tissue)	20.89 ± 1.31^a^	20.82 ± 1.11^a^	10.31 ± 1.81^d^	14.95 ± 1.94^c^	18.05 ± 0.95^b^
	Kidney (nmoL/g tissue)	20.61 ± 2.64^a^	20.83 ± 1.27^a^	10.57 ± 0.91^c^	16.99 ± 1.32^b^	17.52 ± 1.89^b^
**SOD**	Erythrocyte (U/gHb)	20.40 ± 2.91^a^	19.96 ± 1.68^a^	7.31 ± 1.20^c^	13.9 ± 2.02^b^	14.92 ± 2.11^b^
	Liver (U/μg protein)	23.76 ± 2.15^a^	24.40 ± 2.05^a^	6.47 ± 0.77^d^	14.36 ± 1.20^c^	17.80 ± 1.66^b^
	Kidney (U/μg protein)	19.42 ± 2.69^a^	19.15 ± 1.87^a^	6.79 ± 1.06^c^	14.27 ± 1.76^b^	15.02 ± 1.25^b^
**CAT**	Erythrocyte (U/gHb)	14.61 ± 1.54^a^	14.51 ± 1.24^a^	3.69 ± 1.16^c^	7.76 ± 1.34^b^	7.94 ± 1.40^b^
	Liver (U/μg protein)	11.21 ± 1.77^a^	10.98 ± 1.45^a^	1.44 ± 0.47^c^	6.76 ± 0.98^b^	7.29 ± 1.24^b^
	Kidney (U/μg protein)	9.87 ± 1.13^a^	9.45 ± 0.92^a^	1.62 ± 0.72^c^	6.11 ± 2.44^b^	7.04 ± 1.10^b^

*Note*: Mean ± standard deviations; *n*: 6; values with different letters in the same column are statistically significant (*p* < 0.001).

Abbreviations: DF, diclofenac; TA, tubuloside A; SL, silymarin.

### 3.4 Effect on gene expression levels

**FIGURE 3:**
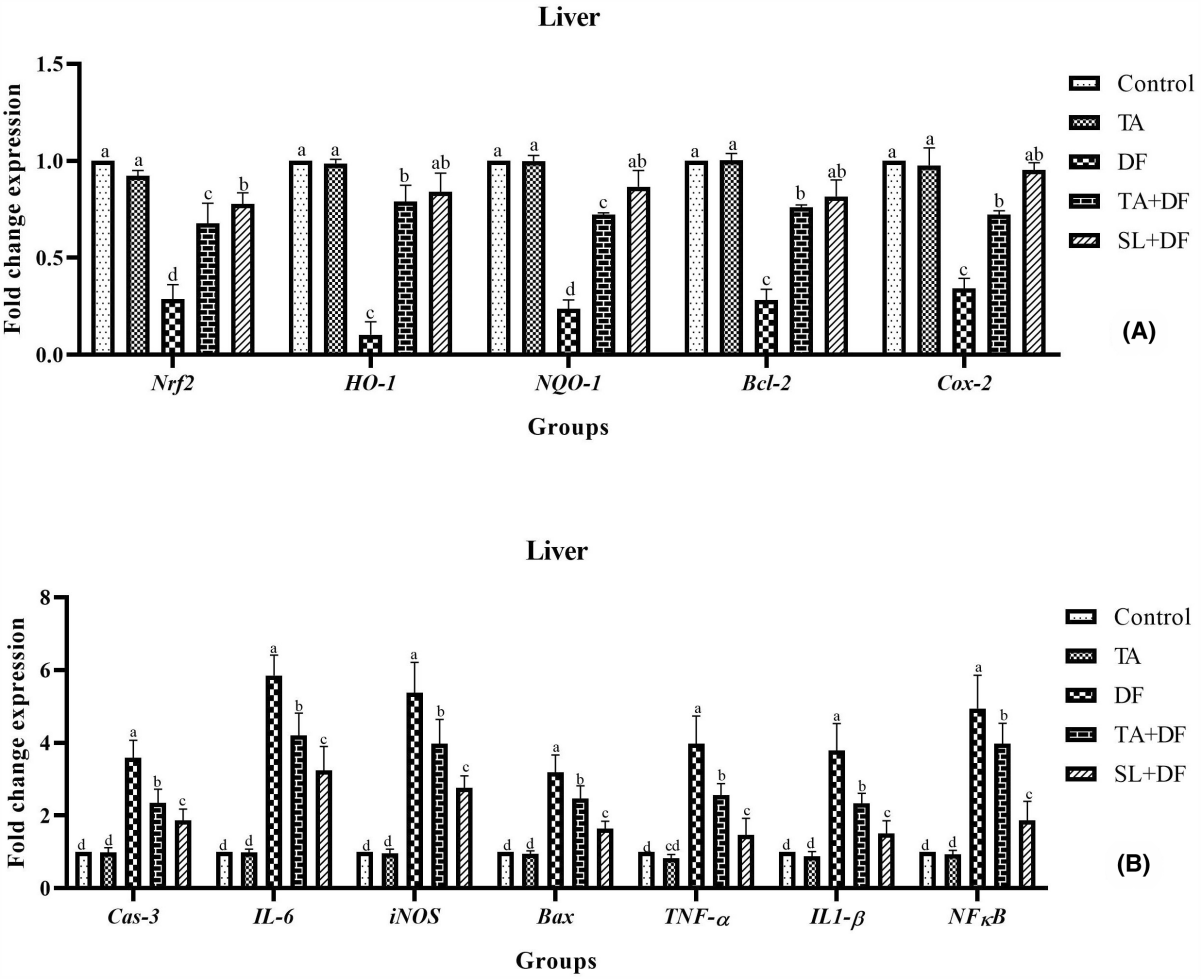
The effect of Diclofenac (DF) and Tubuloside A on mRNA expression levels of *Nrf2*, *HO*‐*1*, *NQO*‐*1*, *Bcl*‐*2* and *Cox*‐*2* (A), as well as *Cas*‐*3*, *IL*‐*6*, *iNOS*, *Bax*, *TNF*‐*α*, *IL1*‐*β* and *NFκB* (B) in the liver. Mean ± standard deviations; *n*: 6; ^a, b, c, d^ values with different letters in the figure are statistically significant (*p* < 0.001). DF, diclofenac; TA, tubuloside A; SL, silymarin.

**FIGURE 4:**
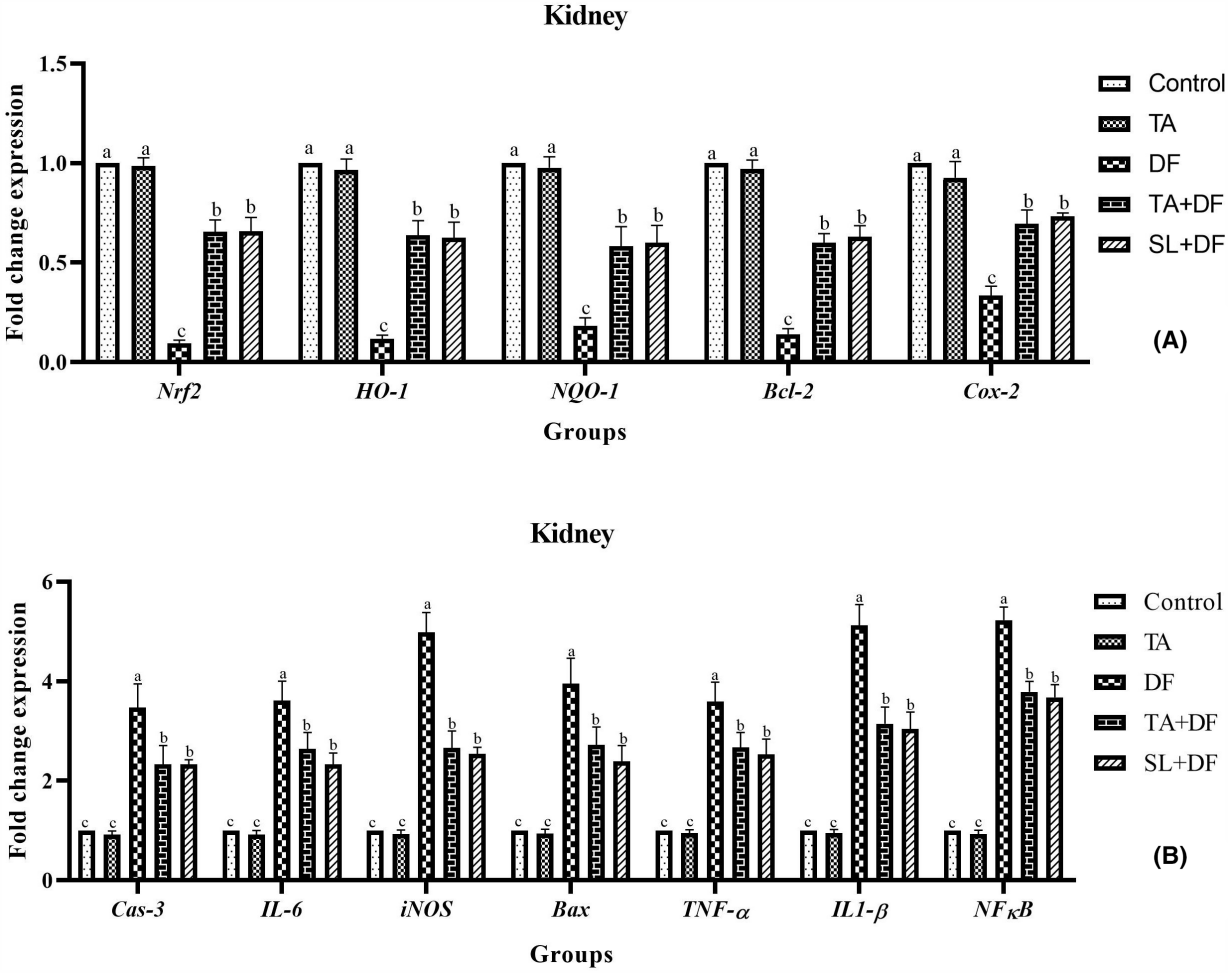
The effect of Diclofenac (DF) and Tubuloside A on mRNA expression levels of *Nrf2*, *HO*‐*1*, *NQO*‐*1*, *Bcl*‐*2* and *Cox*‐*2* (A), as well as *Cas*‐*3*, *IL*‐*6*, *iNOS*, *Bax*, *TNF*‐*α*, *IL1*‐*β* and *NFκB* (B) in the liver. Mean ± standard deviations; *n*: 6; ^a, b, c:^ values with different letters in the figure are statistically significant (*p* < 0.001). DF, diclofenac; TA, tubuloside A; SL, silymarin.

The mRNA expression levels of genes involved in the *Nrf2*/*HO*‐*1* signalling pathway (*Nrf2*, *HO*‐*1*, *NQO*‐*1*, *IL*‐*6*, *iNOS*, *Cox*‐*2*, *TNF*‐*α*, *IL1*‐*β* and *NFκB*) and apoptosis (*Bcl*‐*2*, *Cas*‐*3 and Bax*) in the liver (Figure 3A,B) and kidney (Figure 4A,B) tissues were examined to determine the effects of TA, SL and DF treatments. The analysis revealed that the mRNA expression levels of *Nrf2*, *HO*‐*1*, *NQO*‐*1*, *Cox*‐*2* and *Bcl*‐*2* were downregulated with DF treatment, while the mRNA expression levels of *Cas*‐*3*, *IL*‐*6*, *iNOS*, *Bax*, *TNF*‐*α*, *IL1*‐*β* and *NFκB* were upregulated (*p* < 0.001). TA and SL treatments were found to regulate the mRNA expression changes induced by DF and bring them closer to the control (*p* < 0.001). Additionally, TA treatment alone did not significantly affect these values compared to the control (*p* > 0.05).

### 3.5 Effect on histopathological changes

**FIGURE 5:**
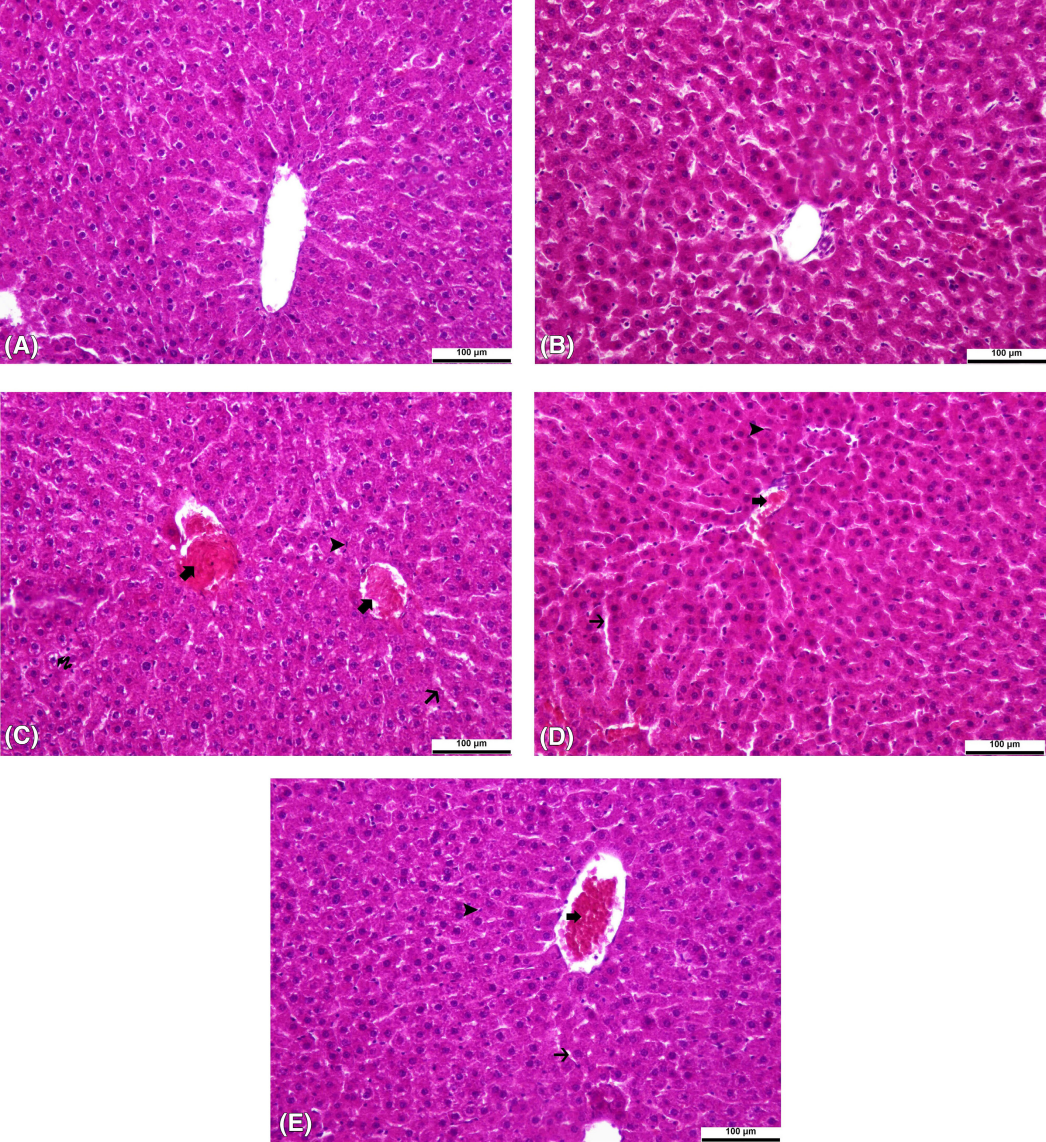
Histopathological changes induced by Diclofenac (DF) and Tubuloside A in the liver tissue of rats. All figures were stained with haematoxylin and eosin. An original magnification of 20× and 100 μm were used. The thick arrow indicates central vein congestion, the thin arrow indicates areas of sinusoidal dilation and congestion, the arrowhead indicates increased Kupffer star cells, and the curved arrow indicates degenerative changes in hepatocytes. (A), (B), (C), (D) and (E) are the control group, TA group, DF group, TA + DF group, and SL + DF group, respectively. DF, diclofenac; TA, tubuloside A; SL, silymarin.

**FIGURE 6:**
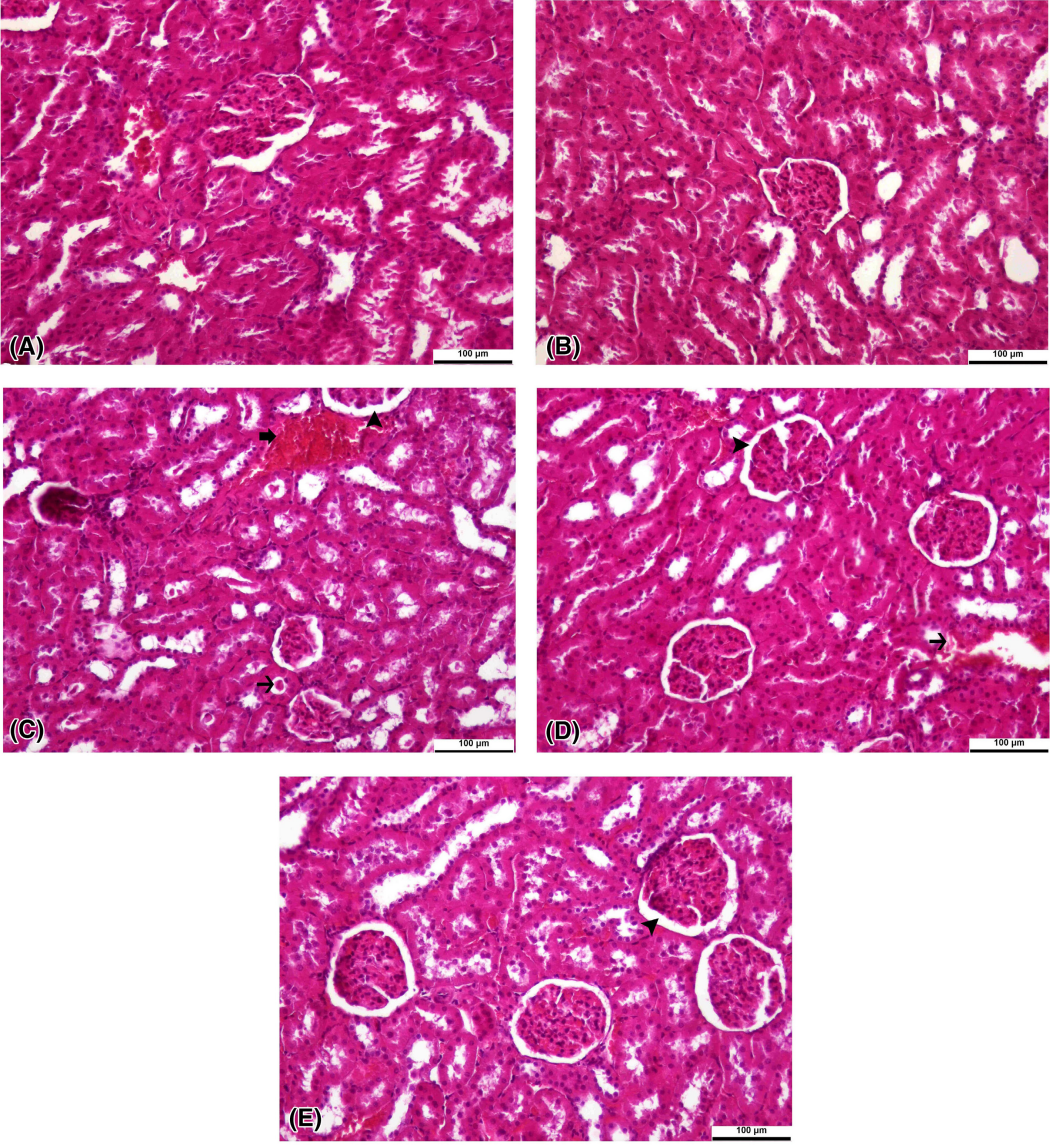
Histopathological changes induced by Diclofenac (DF) and Tubuloside A in the kidney tissue of rats. All figures were stained with haematoxylin and eosin. An original magnification of 20× and 100 μm were used. The thick arrow indicates haemorrhage in the interstitium, the thin arrow indicates hyaline cast formation in the tubular lumen, and the arrowhead indicates the widening of Bowman's space. (A), (B), (C), (D) and (E) are the control group, TA group, DF group, TA + DF group and SL + DF group, respectively. DF, diclofenac; TA, tubuloside A; SL, silymarin.

DF treatment was found to cause central vein hyperemia, sinusoidal dilation with hyperemia areas, an increase in Kupffer star cells, and degenerative changes in hepatocytes in liver tissue (Figure 5C). In the kidney tissue, interstitial haemorrhage, hyaline casts in tubular lumens, and widening of Bowman's space were found as signs of DF toxicity (Figure 6C). TA and SL treatments were observed to reduce the damages caused by DF in the liver (Figure 5D,E) and kidney (Figure 6D,E) tissues. Also, only TA (Figures 5 and 6B) treatment did not cause histopathological changes in tissues compared to the control group (Figures 5 and 6A). In addition, the statistical evaluations of liver and kidney histopathology were expressed in Table 3.

**TABLE 3:** The statistical evaluation of TA and DF administration on histopathological alterations in liver and kidney tissue of rats.

Tissue	Histopathological alterations	Groups				
		Control	TA	DF	TA + DF	SL + DF
Liver	Hyperemia in the vena centralis	0.00 ± 0.00^c^	0.00 ± 0.00^c^	3.66 ± 0.51^a^	2.33 ± 0.52^b^	2.00 ± 0.63^b^
	Sinusoidal dilatation and hyperemia	0.00 ± 0.00^c^	0.00 ± 0.00^c^	3.33 ± 0.50^a^	2.33 ± 0.51^b^	2.30 ± 0.50^b^
	Increase in Kupffer stellate cells	0.00 ± 0.00^c^	0.00 ± 0.00^c^	3.50 ± 0.54^a^	2.30 ± 0.50^b^	1.83 ± 0.70^b^
	Degenerative changes in hepatocytes	0.00 ± 0.00^c^	0.00 ± 0.00^c^	3.66 ± 0.81^a^	2.66 ± 0.80^b^	2.33 ± 0.75^b^
Kidney	Haemorrhage in the interstitial area	0.00 ± 0.00^c^	0.00 ± 0.00^c^	3.33 ± 0.51^a^	2.00 ± 0.70^b^	1.83 ± 0.50^b^
	Hyaline cylinder formations in tubule lumens	0.00 ± 0.00^c^	0.00 ± 0.00^c^	3.00 ± 0.63^a^	2.33 ± 0.51^b^	2.16 ± 0.75^b^
	Enlargement of the Bowman's space	0.00 ± 0.00^c^	0.00 ± 0.00^c^	3.00 ± 0.63^a^	1.83 ± 0.75^b^	1.70 ± 0.70^b^

*Note*: Mean ± standard deviations; *n*: 6; values with different letters in the same column are statistically significant (*p* < 0.001).

Abbreviations: DF, diclofenac; TA, tubuloside A; SL, silymarin.

## 4 DISCUSSION

Depending on the usage, NSAIDs have been reported to cause liver and kidney damage and affect parameters associated with these organs.^29^ It has been reported that treatment of ibuprofen and celecoxib, NSAID drugs, in rats at a dose of 40 mg/kg for 28 days increased liver and kidney function parameters.^30^ Like these drugs, the treatment of DF in rats at a dose of 10 mg/kg intramuscularly for 14 days^31^ and 50 mg/kg intraperitoneally for 2 days^32^ has been reported to increase AST, ALT and ALP activity levels as well as BUN and creatinine levels. Similarly, this study found that DF increased liver enzyme activities and kidney function parameters, whereas TA treatment reduced these values. This suggests that TA exhibited a protective effect on the tissues due to its potent antioxidant activity.

The level of 8‐OHdG, an indicator of oxidative damage, tends to increase depending on the dosage and duration of NSAID drugs.^33^ In a 2‐week clinical study investigating whether geranylgeranylacetone (GGA) protects against DF‐induced gastric mucosal damage, it was reported that 8‐OHdG production increased in the gastric mucosa of humans receiving 75 mg/day DF treatment, while 150 mg/day GGA treatment reduced the DF‐induced increase in 8‐OHdG.^34^ Indomethacin, an NSAID drug, was administered orally to rats at a 25 mg/kg dose, increasing the level of 8‐OHdG in kidney tissue; however, it was reported that oleuropein treatment at different doses (75, 150, and 300 mg/kg) reduced this level.^35^ In our study, parallel to the mentioned studies, TA treatment reduced the increased levels of 8‐OHdG in plasma, liver, and kidney tissues due to DF treatment and decreased oxidative stress, thanks to its antioxidant effect.

Tissue damage induced by drug use is generally triggered by oxidative damage caused by excessively produced free radicals.^36^ In our study, following DF treatment, there was a significant increase in MDA in blood, liver and kidney, as well as a decrease in GSH levels and SOD and CAT activities. These results indicate oxidative damage that leads to an increase in lipid peroxidation and a decrease in antioxidant enzyme activities due to the drug's toxicity. In line with the results of our study, some studies have shown that DF treatment in rats increases lipid peroxidation and reduces the effectiveness of antioxidants.^37^, ^38^, ^39^ However, the significant decrease in MDA and increase in GSH levels and SOD and CAT activities observed with TA treatment demonstrate its regulatory effects against oxidative damage by reducing free radical production. This is consistent with previous reports of the ability of TA, a phenylethanoid compound, to improve tissue antioxidant status in rats exposed to drugs.^40^, ^41^

To investigate the underlying mechanism of TA's protective effect against DF‐induced liver and kidney damage, we determined their effects on *Nrf2*/*HO*‐*1* signalling and inflammatory mediators. It was observed that DF treatment in rats downregulated *Nrf2*, *HO*‐*1* and *NQO*‐*1* mRNA expression levels. *Nrf2*, a transcription factor associated with genes encoding antioxidants, detoxifying enzymes, and related stress‐sensitive proteins, protects cells against conditions that cause oxidative damage, such as inflammation, apoptosis and carcinogenesis.^42^ *Nrf2* regulates antioxidants such as SOD and CAT and cytokines such as *NQO*‐*1* and *HO*‐*1*.^43^ Karimi‐Matloub et al.^44^ found that *Nrf2* and *HO*‐*1* expression levels were downregulated in kidney tissue after intraperitoneal treatment of 50 mg/kg/7 days DF in rats and that elagic acid treatment (10 mg/kg/7 days) upregulated these values. In addition, it was concluded that suppression of *HO*‐*1* occurs in liver damage induced by lipopolysaccharide/DF, and this can be upregulated by curcumin and/or selenium treatment, thus playing a role in alleviating oxidative stress and inflammation.^45^ In addition, DF treatment reduced the expression levels of *Cox*‐*2* and *Bcl*‐*2* genes, which are effective in inflammation and apoptotic processes, whereas the mRNA expression levels of *Cas*‐*3*, *IL*‐*6*, *iNOS*, *Bax*, *TNF*‐*α*, *IL1*‐*β* and *NFκB* were significantly increased. Here, DF induces an inflammatory response by activating the *NF*‐*κB* pathway and then increasing the release of inflammatory mediators. In this context, it has been revealed that DF treatment causes an increase in the levels of *NF*‐*κB*,^46^ *IL*‐*1β* ^37^ and *TNF*‐*α*,^47^ which are effective in inflammation, and the levels of *Cas*‐*3* and *Bax*, which are effective in the apoptotic process, while causing a decrease in the expression of the anti‐apoptotic gene *Bcl*‐*2* ^39^ and *Cox*‐*2* expression^48^ in the liver and kidney tissues of rats. On the other hand, TA supplementation has prevented increased inflammation and apoptosis with DF on the *Nrf2*/*HO*‐*1* signal. These findings have shown that TA plays a central role in the hepato‐renal protective effect against DF. In support of these findings, it has been reported that *C*. *tubulosa* containing phenylethanoid oligoglycosides such as TA showed hepatoprotective effects against d‐galactosamine d‐GaIN)/lipopolysaccharide‐induced liver damage in mice.^15^

It has been reported that DF causes fatty changes, hyperemia, cytoplasmic vacuolation, and degenerative changes in hepatocytes in the livers of rats, and large fibrotic areas and cell apoptosis in kidney tissue.^48^ Similarly, in a study by Elshopakey and Elazab,^3^ DF treatment was reported to cause dilatation in the sinusoids of rat liver tissue, necrosis in hepatocytes, the proliferation of renal glomeruli, and the presence of significant bleeding areas in the interstitial tissue of the kidney. In our study, similar histopathological findings were observed regarding the damage that occurred in the liver and kidney tissues as a result of DF treatment, while TA treatment reduced the damage caused by DF and exhibited cytoprotective effects.

As a result, DF created oxidative stress on the liver and kidney by increasing the *Nrf2*/*HO*‐*1* signal and releasing inflammatory mediators, while TA prevented this situation. This situation showed that clinically, TA might potentially be used to reduce hepato‐renal toxicity caused by DF.

## AUTHOR CONTRIBUTIONS

**Ali Tureyen:** Conceptualization (equal); methodology (equal); supervision (equal); visualization (equal); writing – original draft (equal); writing – review and editing (equal). **Hasan Huseyin Demirel:** Data curation (equal); investigation (equal); methodology (equal). **Ezgi Nur Demirkapi:** Investigation (equal); methodology (equal). **Azra Eryavuz:** Investigation (equal); methodology (equal). **Sinan Ince:** Data curation (equal); investigation (equal); methodology (equal); writing – review and editing (equal).

## CONFLICT OF INTEREST STATEMENT

The authors confirm that there are no conflicts of interest.

## Data Availability

The data that support the findings of this study are available from the corresponding author upon reasonable request. All data generated or analysed during this study are included in the manuscript.
